# Current trends and challenges on dementia management and research in Latin America

**DOI:** 10.7189/jogh.10.010362

**Published:** 2020-06

**Authors:** Ricardo Nitrini, Maira Tonidandel Barbosa, Sonia Maria Dozzi Brucki, Mônica Sanches Yassuda, Paulo Caramelli

**Affiliations:** 1 Departamento de Neurologia, Faculdade de Medicina da Universidade de São Paulo, São Paulo, São Paulo, Brazil; 2 Departamento de Clínica Médica, Faculdade de Medicina da Universidade Federal de Minas Gerais, Belo Horizonte, Minas Gerais, Brazil; 3 Faculdade de Ciências Médicas de Minas Gerais, Belo Horizonte, Minas Gerais, Brazil; 4 Escola de Artes, Ciências e Humanidades da Universidade de São Paulo, São Paulo, São Paulo, Brazil

This manuscript presents an overview of the current scenario and challenges on dementia practice and research in Latin America (LA). We review the main demographic and socioeconomic indicators from LA, epidemiology and diagnosis of dementia, knowledge of dementia by the general public, by general practitioners (GPs) and by other health care professionals, costs of dementia, and the research on dementia in the continent. The main aim of this article is to integrate information regarding some of the action areas aligned by the World Health Organization (WHO, 2017) global action plan on the public health response to dementia [1], which will need to be addressed by the countries in the region, as they prepare their local dementia action plans.

## MAIN DEMOGRAPHIC AND SOCIOECONOMIC CHARACTERISTICS

LA is constituted by territories where mainly the Spanish or Portuguese languages are spoken today, from Mexico in the north, to Argentina and Chile in the south, including Central and South America, as well as many Caribbean countries.

A recent publication of the Pan American Health Organization [2] compares area and demographic indicators from LA to Canada and the USA. Although the geographic area is quite similar, the population of the region is 1.8 times greater than that of Canada and the USA combined. The population growth per year is reducing in both regions, but the number of older adults (65+ years) is increasing at a faster pace in LA, representing 9.0% of the total population nowadays, due to a rapid advance of life expectancy (**Table 1**).

**Table 1:** Area and demographic indicators of Latin America and Canada/USA (2018)*

	Latin America	Canada + USA
Area	19 197 000 km^2^	19 509 737 km^2^
Population	644 481 000	363 721 000
Population growth/y	1.0%	0.7%
Population aged 65+ years	9.0%	16.0%
Life expectancy at birth	76 y	80 y

*Source: Pan American Health Organization [2].

In relation to socioeconomic indicators, the same report states that the mean educational level is 8.3 years in LA, compared to 13.5 years in Canada and the USA. The gross national income per capita is three times higher in the USA and around four times higher in Canada than in LA. The gross domestic product (GDP) growth was 1.7% in the region in 2017, compared to 2.3% and 3.0% in USA and Canada, respectively. Health expenses, expressed as percentage of annual GDP, are 60% lower in LA, according to 2015 data, both in public and private sectors (**Table 2**).

**Table 2:** Socioeconomic indicators in Latin America and Canada/USA*

	Latin America	Canada + USA
Mean years of schooling (2015)	8.3	13.5
Gross National Income ppp US$ per capita (2017)	15 288	Canada: 45 750
		USA: 60 200
Annual GDP growth (2017)	1.7%	Canada: 3.0%
		USA: 2.3%
National health expenditure as % of GDP (2015)	Public: 3.6	Public: 8.5
	Private: 3.4	Private: 7.8

ppp – purchasing power parity, GDP – gross domestic product

*Source: Pan American Health Organization [2].

The distribution of income in LA is strikingly unequal. GINI is an index that measures the extent to which the distribution of income, among individuals within an economy, deviates from a perfectly equal distribution. A GINI index of zero represents perfect equality, while an index of 100 implies complete inequality. Fifteen LA countries are among the 40 nations with the worst GINI in the world [3].

Prevalence of cardiovascular risk factors is higher in LA when compared to Canada and USA. Age-adjusted prevalence rates of high blood pressure are 23.7% among men and 18.1% among women in LA, dropping to 15.3% and 10.5% among men and women, respectively, in Canada and the USA. In these latter countries, the prevalence of diabetes or raised fasting blood glucose is 8.0% in men and 6.2% in women, while in LA these figures are higher, in both men (8.9%) and women (9.6%). On the other hand, tobacco smoking and alcohol consumption are lower in LA than in Canada/USA, namely, 15.3% vs 21.2% and 6.9% vs 9.7%, respectively [2].

## EPIDEMIOLOGY OF DEMENTIA IN LA

Several population-based studies, as well as some systematic reviews, meta-analyses and opinion papers on dementia in LA have been published in the last 25 to 30 years [4-14]. These studies have shown that dementia is an emergent giant problem for LA.

A review of eight population-based investigations from six different countries found a crude prevalence of 7.1% (95% confidence interval (CI) = 6.8-7.4) of dementia in individuals aged 65+ years [8]. This rate was comparable with the prevalence observed in developed countries, although among the higher rates previously reported. Subsequently, a systematic review and meta-analysis [9] estimated that dementia prevalence in the region was 8.5% among individuals aged 60+ years, representing the highest rate in the world. In a more recent systematic review, Zurique-Sánchez et al. reported a prevalence of dementia in LA of 11.5% in the population aged 65+ years, with dementia being more common in women and in urban areas [10].

Epidemiological studies on the prevalence of dementia in Latin America have been performed in several countries and have shown large variations in prevalence rates, such as prevalence rate of 2.0% in a Brazilian population study[11] and of 13.1% in a Venezuelan population study [12], both including individuals aged 65+ years. These differences are probably more related to methodological variability, from the diagnostic criteria of dementia to definitions of cognitive and functional impairment, than to cultural, social, environmental or genetic conditions of each country [15]. Even when methods are harmonized, differences in prevalence rates may be observed across countries in the region. In one population-based cross-sectional survey, which included five LA countries and used the 10/66 Dementia Research Group diagnostic algorithm, the prevalence of dementia ranged from 6.2% in Venezuela to 12.6% in Cuba for individuals aged 65 + years, and the response proportion was at least 80% [5]. However, heterogeneity in prevalence rate data are also common in studies from other regions of the world, as described by a meta-analysis of the global prevalence of dementia, where heterogeneity of prevalence was higher in South Asia, Western Europe and Asia Pacific than in LA [9].

**Figure:**
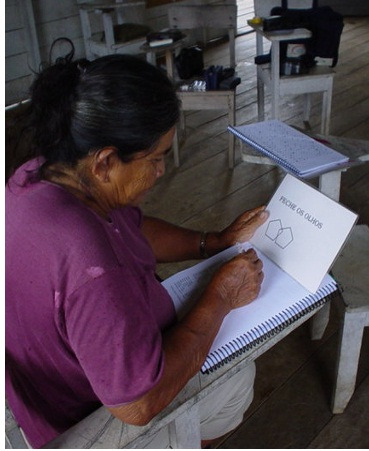
Photo: from the collection of Dr Sonia Brucki (used with permission).

The high prevalence of dementia represents a considerable challenge to the local health and economic systems and this scenario may be worse as the numbers of people living with dementia will have increased 4-fold in LA from 2015 to 2050 [16].

Dementia has been associated with low education and poor cardiovascular health in prevalence studies, but there are only a few incidence studies in LA to evaluate risk factors [17-20]. In these studies, incidence was similar [17,19] or higher in LA countries than in Western countries [18]. Besides age, other risk factors included markers of low cognitive reserve [18], poor cardiovascular health [20], and being a carrier of at least one APOE-ϵ 4 allele [19].

There is an urgent need to ensure some level of methodological harmonization among researchers when performing epidemiological studies on dementia in LA, to include more geographical areas in each country and to have more studies on incidence and mortality rates [15]. Joint efforts of clinicians and epidemiologists would be essential to improve the quality of epidemiological data on dementia in LA.

Besides the high overall prevalence of dementia reported in most studies, prevalence among younger elderly (ie, 65-69 years) was found to be 2.4%, exactly twice the rate reported by a systematic review of 21 studies conducted in Europe [8]. In addition, two studies that were more recently conducted in Argentina [13] and in Brazil [14], including individuals with 60+ years, also found higher rates within this age range in comparison to North America and Western European countries.

These numbers suggest that dementia may start earlier in the region. However, plausible explanations for this finding are challenging. These figures could represent a bias, due to difficulties in diagnosis of dementia. Alternatively, environmental factors could account for these features.

### Environmental factors

Low educational level is a well-known risk factor for dementia. It is usually associated with low socioeconomic status and with reduced access to health care, from the prenatal period to advanced ages. Hence, low education might increase dementia risk by limiting adequate diagnosis and treatment of comorbidities, particularly cardiovascular diseases and diabetes mellitus, as well as being commonly associated with impaired nutritional status. Moreover, low educational attainment is also associated with low cognitive reserve [21], thus possibly leading to the earlier manifestation of dementia symptoms. In the collaborative study on prevalence of dementia in six LA countries, mentioned above, the prevalence among illiterate elderly was twice the rate among literate individuals, namely 15.7% *vs*. 7.2% [8].

Illiteracy in the adult population is not particularly high in LA. In fact, the average literacy rate is 94% in the continent [22]. However, the illiteracy rate among the elderly population is high, currently at 21%. Moreover, at least in Brazil, there is an unequal distribution of literacy rates, with lower figures in specific regions (North and Northeast) and rural areas of the country. Hence, low education and illiteracy are key issues affecting the lives of older people in LA.

Besides formal illiteracy, another problem is functional illiteracy. Functional illiterates are able to read and to write short sentences, but they are unable to use written language – including numbers – to deal with the daily-life requirements. In a Brazilian study, 312 healthy individuals with mean age of 47.3 years and mean educational level of 9.7 years (ranging from 1-17 years) were evaluated with a test of functional health literacy. Functional illiteracy was found in 32.4% of the sample. Of note, only a small proportion of individuals with one to seven years of schooling performed in the adequate range [23].

Another relevant issue is the high presence of indigenous people in LA. In 2010, there were 42 million indigenous people living in the continent, corresponding to 8% of the total population. About 34 million (80.1%) live in Bolivia, Guatemala, Mexico and Peru. Most of these individuals live in urban areas and are bilingual, but their educational level is lower than other groups [24].

So far, we do not have a large number of studies on cognitive impairment and dementia in the indigenous populations of LA. In a Colombian study, Moreno et al [25] found that Native American ancestry was associated with lower risk of late-onset Alzheimer disease (AD), while African ancestry individuals displayed a higher risk. In a systematic review that included Australia, Brazil, Canada, Guam and the USA, [26], the prevalence of dementia among indigenous populations appears to be higher than in non-indigenous populations, but there is a paucity of high quality epidemiological research regarding this issue. In another systematic review, it was suggested that indigenous people may be more vulnerable to cognitive disorders, due to low cognitive reserve and exposure to poor health status throughout the life span [27]. Indeed, a survey of 217 indigenous individuals living in in the Brazilian Amazon found high rates of cognitive impairment and dementia, namely 43.0% in individuals aged 50+ years and 51.1% in those aged 65+ years [28]. Of note, in this study, cognitive impairment was assessed with a culturally adapted instrument.

In summary, the demographic and clinical factors that may be responsible for the increased prevalence of dementia in LA are low education (leading to low cognitive reserve), low socioeconomic level (contributing to low brain reserve), low health expenditures as percentage of GDP in LA countries, high rates of high blood pressure and raised blood glucose levels/diabetes. In relation to the latter issue, two neuropathological studies from a Brazilian brain bank have shown high rates of vascular changes among elderly subjects. Indeed, the prevalences of vascular dementia in these two studies were higher than those that have been reported in developed countries [29,30].

It is important to note that the factors listed above are all modifiable and thus, cognitive impairment and dementia related to these aspects may be preventable. In 2017, Livingston et al. [31] suggested that nine potentially modifiable risk factors (less childhood education, midlife hearing loss, hypertension and obesity, later-life smoking, depression, physical inactivity, social isolation, and diabetes) account for 35% of worldwide dementia cases. In 2019, using similar analytical strategies, Mukadam et al [32] indicated that five dementia risk factors were more prevalent in low- and middle-income countries (LMIC) than worldwide estimates, such as less childhood education, smoking, hypertension, obesity, and diabetes. Estimates presented in the study suggested that 56% of dementia cases in LA (using data from Cuba, Dominican Republic, Mexico, Peru, Puerto Rico, and Venezuela) are accounted by such modifiable factors [24]. Therefore, the dementia prevention potential in the region is larger than in high-income countries.

### Difficulties in the diagnosis of dementia

Higher prevalence of dementia may also be explained by difficulties in the diagnosis. The use of paper and pencil tests, developed in high-income countries, to detect cognitive impairment in low educated individuals may lead to an elevated number of false positive cases, even with education-adjusted cut-off scores. To attenuate this bias, several screening tests, such as the Mini-Mental State Examination (MMSE) [33], Montreal Cognitive Assessment test [34], Clock Drawing Test [35], and verbal fluency measures [35] have been culturally adapted and validated and are widely used in the region with adjusted cut-off scores in research and in clinical settings. Similarly, global cognitive batteries, such as the Mattis Dementia Rating Scale [36] and the Addenbrooke’s Cognitive Examination-Revised [37], are also used.

On the other hand, there are cognitive tests proposed for multicultural populations which are expected to be less influenced by education, for instance, the Consortium to Establish a Registry for Alzheimer Disease [38], Rivermead Behavioural Memory Test [39], Fuld Object Memory Evaluation [40], Short-term Memory Binding Test [41], Rowland Universal Dementia Assessment Scale [42], Neuropsi battery [43], Brief Cognitive Screening Battery [44,45] and the Stick Design Test [46] which have been used in LA studies. For recent reviews of cognitive screening studies conducted in low literacy settings, see Ortega et al [47] and Paddick et al. [48].

Another important strategy that may contribute to dementia detection in LA is the association of brief cognitive tests with questionnaires of functional activities applied to informants. Instruments, such as the Pfeffer Functional Activities Questionnaire (PFAQ) [49], have been used in combination with cognitive tests, such as the MMSE, in population-based studies on dementia in Brazil, Chile and Peru [50-52]. Other previously used instruments are the Informant Questionnaire on Cognitive Decline in the Elderly [53], the AD8 [54] and the Cognitive Change Questionnaire-8 [55].

We shall consider that diagnostic bias is not a probable explanation for the dementia prevalence figures in LA. Several studies conducted in various LA countries with different methods and tests have shown a high prevalence of dementia. Moreover, studies performed in different LA countries and abroad, using similar methods, confirmed the high prevalence of dementia in LA [5,9].

If a conservative prevalence of 10% among individuals aged 65+ years is taken into account, and that 9% of the entire population of the region is within this age range, the estimated total number of elderly individuals with dementia in LA would be 5.8 million. With the estimation that the number of people living with dementia will nearly double every 20 years [16], LA countries should be prepared for this huge burden [3,16].

## PUBLIC AWARENESS AND KNOWLEDGE ABOUT DEMENTIA AND AD

Few studies so far have investigated knowledge and public awareness of dementia in LA countries. In a recent study conducted in Brazil, 1414 individuals were interviewed and the researchers observed a very low level of information about AD, even among relatives and caregivers of demented patients [56]. In an editorial comment on this work, Lawlor [57] stated that it is imperative to raise public awareness of dementia in the region.

In this sense, in LA it is vital to make efforts to increase awareness about dementia and its most common early simptoms in order to increase diagnosis and timely treatment, to reduce stigma, to treat modifiable risk factors and increase the potential for dementia prevention in adult life. These features may increase bottom-up effects on policy makers, as society may demand change in current practice and settings to accommodate dementia care [58]. We definetly need more studies regarding dementia awareness in LA, with follow-up data collection after campaigns, to assess efficacy [56].

Another important issue is knowledge of dementia by GPs. In LA, the diagnosis is usually made by specialists and sporadically by a GP [4], in contrast to usual procedures in many European countries, where most patients with dementia are diagnosed by a GP [59]. We shall acknowledge that medical conditions with very high prevalence must be primarily diagnosed and treated by GPs, such as high blood pressure, diabetes mellitus, headache, depression and also dementia.

In a study conducted in Brazil, 248 randomly selected elderly patients seen in a GP outpatient clinic from a university hospital were submitted to cognitive and functional evaluations. Among subjects with confirmed cognitive impairment, only 16.3% had information on cognitive complaints or decline in their medical files [60]. These numbers represent a very low rate of identification of cognitive impairment by GPs, in comparison to what is found in Europe and North America (20% to 65%) [61,62].

Within this scenario, strategies to increase knowledge on dementia among GPs and health care providers are needed. Information on dementia should be included even in elementary, middle and high school. It should also be explored in more detail in medical schools and in graduate courses of other health disciplines in our continent. GPs need to actively investigate whether her/his patient has cognitive decline, to prescribe treatment and to orient healthier behavior [59]. It is also essential to combat nihilism and to advocate that changes in adult life and in relatively younger elderly may contribute to prevent dementia [63,64].

Finally, it is necessary to show that it is possible to easily screen for dementia. Indeed, brief and accessible instruments for cognitive and functional evaluation are available for use by GPs in most LA countries. For example, the combination of PFAQ (>2) and verbal fluency test (<10 animals/min.) displayed 88.3% of sensitivity and 76.5% of specificity for the detection of cognitive impairment [65]. Category fluency (animals) is a one-minute task, which has been proven to be sensitive and specific for AD dementia diagnosis in populations with variable degrees of schooling, from illiterates to university-level. Indeed, in a recent study conducted in the USA, category fluency (animals in one minute) did not differ from either the MMSE or Memory Impairment Screen in relation to diagnostic accuracy [66]. Therefore, there should be a call for action among health professionals, especially in primary care.

## COSTS OF DEMENTIA

Research on dementia costs have been performed in Argentina [67], Brazil [68,69], Chile [70-72] and Peru [73]. Also, studies of the 10/66 Dementia Research Group in LMIC included data from Cuba, Dominican Republic, Mexico, Peru and Venezuela [74,75]. The available data suggest that costs increase with the severity of the dementia, as reported in two studies (67,73] and indirect (informal) costs predominate in LA [69,70].

In high-income countries, direct costs (professional care in the community, and the costs of residential and nursing home care) account for most of the expenses (16, 76,77]. In many LA countries, a few direct costs (doctors, medicines) are paid by the public health system. However, formal caregivers, home-based meal programs, institutionalization in residential and nursing home care or support to family caregivers are not generally available [6,16,69].

The costs of dementia in LMIC are much lower when compared to high-income countries [76,77]. Costs of dementia for upper-middle income countries is approximately US$ 5284.00 per person per month [76]. In Chile, the monthly cost was higher for low SES (US$ 1,588) than for high SES (US$ 1,083), but most (74%) of it is spent in informal care [70]. In Brazil, the cost of dementia was estimated at US $ 1405.72 per person per month, with 56.6% attributable to informal care costs (costs associated with caregiver productivity loss and time spent by caregiver on patient support) [69]. Informal care costs are afforded by the families due to lack of social / public support. Although the estimated cost does not seem high, informal care represents a significant financial burden, as the average family income tends to be very low. For instance, the costs of informal care reported by Ferretti et al. [69] represent more than 3 times the national minimum wage.

Among the family caregivers, women perform most of the tasks [78,79]. When they cannot leave their job, families tend to hire untrained and inexperienced women to undertake poorly paid and unsupervised care-work with no social support [74]. Informal care is not an exclusive problem of LMIC as it is a significant challenge for high-income countries too (16). However, in LMIC, many people with dementia face several other overlapping disadvantages, such as low education, low or no income, lack of pension or health insurance [75].

With both the expected increase in the number of dementia cases due to aging of the population [80] and reduction in the number of members of the urban families in LA [81], informal care will be even more difficult in the near future and it is already one of the main challenges that need to be faced by the society and policy makers in the region.

As it was proposed for prevalence and incidence studies, strategies for the evaluation of costs in dementia need to be harmonized for regions within each country and across countries in LA. Hamonization efforts should reveal the real burden of dementia in the region and it should aid policy makers to plan the ideal funds to meet the health needs of the population [15].

## RESEARCH ON DEMENTIA IN LA

Undoubtedly, conducting research in LA is quite challenging. There is a clear lack or limited availability of public and private grants. In spite of this, the total number of publications related to dementia carried out in LA and indexed in PubMed increased five times in the last 10 years, representing 3.35% of the papers indexed in that period. In the Web of Science platform, the total number of publications increased 2.5 times in 10 years, representing 3.71% of the indexed papers on this topic in that period.

Although there is a significant amount of research activity in dementia carried out in LA, much more public funding is needed to attend to the key priority issues, as established by the WHO global action plan [1]. In addition, regional collaborative research is needed, based on harmonized protocols and with data sharing.

## CONCLUSIONS

Dementia is an alarming emergent problem for LA. The prevalence of dementia in LA is one of the highest in the world and, with the expected aging of the population, the number of cases of dementia tends to increase 4-fold from 2015 to 2050.

More epidemiological studies on dementia prevalence are needed in several regions in each country and in all LA countries, using harmonized methods to assess the actual prevalence and the evolution of the prevalence rates in the near future. Incidence studies are also needed to identify risk factors and the evolution of incidence after the control of such factors. Harmonized methods for epidemiological studies should unite clinicians and epidemiologists.

Dementia manifests earlier in LA. Prevalence of dementia is higher among illiterate and low educated individuals. The higher prevalence and its earlier emergence are probably related to low cognitive reserve (including low brain reserve), vascular diseases and other potentially modifiable factors.

Diagnosis of dementia among low educated individuals is challenging but may be feasible with questionnaires and cognitive tests less biased by education. Research and sharing of experiences among LA experts on dementia will be important to reach a consensus regarding common or similar methods based on questionnaires and short cognitive tests for diagnosis of dementia by the general practitioner, who cares for most of the elderly population in LA.

Knowledge about dementia should be increased among lay people using public health awareness campaigns to promote early diagnosis and better support after diagnosis. Information on strategies to reduce dementia risk by changing life-style risk factors should be a significant part of these campaigns. Participation of people living with dementia should be stimulated.

Reducing stigma is also a very important issue. The creation of friendly communities for people with dementia should be encouraged.

The costs of dementia care should not be paid solely by families. Local governments need to increase the funding and create support programs to enable better care, including policies related to employment, health and insurance for people living with dementia and their caregivers. Costs of dementia need to be evaluated in every country using harmonized methods to plan funds to meet the health needs of the people living with dementia and their caregivers.

National Dementia Plans have been implemented in a few LA countries. Exchanges of experiences among the countries who have such plans and also with other LA countries are needed to understand the successes and failures of each plan.

It will be necessary to increase research funding from local governments or international agencies to foster local and collaborative studies among LA countries.

A higher synergy among LA countries is needed for information integration and coordinated actions, as countries in the region stride to address the challenges associated to the expected increase in dementia prevalence rates.

The potential for dementia risk reduction in LA seems larger than in high-income countries, therefore, regional collaborative efforts should also focus on prevention. Interventions conducted locally should test strategies aiming to change lifestyle-related risk factors, such as physical inactivity, obesity, unbalanced diets, harmful use of tobacco and alcohol, and the control of diabetes mellitus and midlife hypertension. In addition, interventions based on LA culture and experiences should be devised aiming to reduce social isolation, cognitive inactivity and mid-life depression. Finally, dementia prevention starts in early childhood, as low education is perhaps the most important modifiable dementia risk factor. Therefore, in LA, significant investments in high quality education throughout the life cycle should be a first order priority.

## References

[1] World Health Organization. Global action plan on the public health response to dementia 2017 – 2025; 2017. Available: https://www.who.int/mental_health/neurology/dementia/action_plan_2017_2025/en/. Accessed: 19 October 2019.

[2] Pan American Health Organization. Health Situation in the America: Core Indicators 2018. Available: https://iris.paho.org/bitstream/handle/10665.2/49511/CoreIndicators2018_eng.pdf?sequence=1&isAllowed=y. Accessed: 19 October 2019.

[3] Index Mundi. Gini Index. World Bank Estimates. Country ranking. Available: https://www.indexmundi.com/facts/indicators/SI.POV.GINI/rankings. Accessed: 19 October 2019.

[4] Manes F The huge burden of dementia in Latin America. Lancet Neurol. 2016;15:29. 10.1016/S1474-4422(15)00360-9 26700903

[5] Llibre Rodriguez JJ Ferri CP Acosta D Guerra M Huang Y Jacob KS Dementia Research Group. Prevalence of dementia in Latin America, India, and China: a population-based cross-sectional survey. Lancet. 2008;372:464-74. 10.1016/S0140-6736(08)61002-8 18657855 PMC2854470

[6] Custodio N Wheelock A Thumala D Slachevsky A Dementia in Latin America: epidemiological evidence and implications for public policy. Front Aging Neurosci. 2017;9:221. 10.3389/fnagi.2017.00221 28751861 PMC5508025

[7] Parra MA Baez S Allegri R Nitrini R Lopera F Slachevsky A Dementia in Latin America. Assessing the present and envisioning the future. Neurology. 2018;90:222-31. 10.1212/WNL.0000000000004897 29305437 PMC5791795

[8] Nitrini R Bottino CM Albala C Custodio Capuñay NS Ketzoian C Llibre Rodriguez JJ Prevalence of dementia in Latin America: a collaborative study of population-based cohorts. Int Psychogeriatr. 2009;21:622-30. . 10.1017/S1041610209009430 19505354 PMC8324310

[9] Prince M Bryce R Albanese E Wimo A Ribeiro W Ferri CP The global prevalence of dementia: a systematic review and metaanalysis. Alzheimers Dement. 2013;9:63-75.e2. 10.1016/j.jalz.2012.11.007 23305823

[10] Zurique Sánchez CZ Sanabria MOC Sánchez MZ López PAC Sánchez MS Hernández SH Prevalence of dementia in the elderly in Latin America: a systematic review. Rev Esp Geriatr Gerontol. 2019;S0211-139X(19)30011-3. 10.1016/j.regg.2018.12.007 30772072

[11] Ramos-Cerqueira AT Torres AR Crepaldi AL Oliveira NI Scazufca M Menezes PR Identification of dementia cases in the community: a Brazilian experience. J Am Geriatr Soc. 2005;53:1738-42. . 10.1111/j.1532-5415.2005.53553.x 16181173

[12] Maestre GE Pino-Ramírez G Molero AE Silva ER Zambrano R Falque L The Maracaibo Aging Study: population and methodological issues. Neuroepidemiology. 2002;21:194-201. 10.1159/000059524 12065882

[13] Bartoloni L Blatt G Insua I Furman M González MA Hermann B A population-based study of cognitive impairment in socially vulnerable adults in Argentina. The Matanza Riachuelo study preliminary results. Dement Neuropsychol. 2014;8:339-44. 10.1590/S1980-57642014DN84000006 29213923 PMC5619181

[14] César KG Brucki SM Takada LT Nascimento LF Gomes CM Almeida MC Prevalence of cognitive impairment without dementia and dementia in Tremembé, Brazil. Alzheimer Dis Assoc Disord. 2016;30:264-71. 10.1097/WAD.0000000000000122 26629676

[15] Ferri CP Oliveira D Harmonization of epidemiological studies on dementia in Latin America. Why does it matter? Dement Neuropsychol. 2019;13:363-6. 10.1590/1980-57642018dn13-040001 31844488 PMC6907694

[16] Prince M, Guerchet M, Prina M. The Epidemiology and Impact of Dementia - Current State and Future Trends. WHO Thematic Briefing, 2015. Available: http://www.who.int/mental_health/neurology/dementia/thematic_briefs_dementia/en/. Accessed: 19 October 2019.

[17] Nitrini R Caramelli P Herrera E Jr Bahia VS Caixeta LF Radanovic M Incidence of dementia in a community-dwelling Brazilian population. Alzheimer Dis Assoc Disord. 2004;18:241-6. 15592138

[18] Prince M Acosta D Ferri CP Guerra M Huang Y Llibre Rodriguez JJ Dementia incidence and mortality in middle-income countries, and associations with indicators of cognitive reserve: a 10/66 Dementia Research Group population-based cohort study. Lancet. 2012;380:50-8. 10.1016/S0140-6736(12)60399-7 22626851 PMC3525981

[19] Maestre GE Mena LJ Melgarejo JD Aguirre-Acevedo DC Pino-Ramírez G Urribarrí M Incidence of dementia in elderly Latin Americans: Results of the Maracaibo Aging Study. Alzheimers Dement. 2018;14:140-7. 10.1016/j.jalz.2017.06.2636 28943198 PMC5803319

[20] Perales-Puchalt J Vidoni ML Llibre Rodríguez J Vidoni ED Billinger S Burns J Cardiovascular health and dementia incidence among older adults in Latin America: Results from the 10/66 study. Int J Geriatr Psychiatry. 2019;34:1041-9. 10.1002/gps.5107 30908765 PMC6579616

[21] Stern Y Arenaza-Urquijo EM Bartrés-Faz D Belleville S Cantilon M Chetelat G Whitepaper: Defining and investigating cognitive reserve, brain reserve, and brain maintenance. Alzheimers Dement. 2018;S1552-5260(18)33491-5. 10.1016/j.jalz.2018.07.219 30222945 PMC6417987

[22] UNESCO. Literacy Rates Continue to Rise from One Generation to the Next. UIS Fact Sheet No. 45 | September 2017. Available: http://uis.unesco.org/sites/default/files/documents/fs45-literacy-rates-continue-rise-generation-to-next-en-2017_0.pdf. Accessed: 19 October 2019.

[23] Carthery-Goulart MT Anghinah R Areza-Fegyveres R Bahia VS Brucki SMD Damin A Performance of a Brazilian population on the test of functional health literacy in adults. Rev Saude Publica. 2009;43:631-8. 10.1590/S0034-89102009005000031 19488667

[24] The World Bank. Indigenous Latin America in the Twenty-First Century;2018. Available: https://www.worldbank.org/en/region/lac/brief/indigenous-latin-america-in-the-twenty-first-century-brief-report-page. Accessed: 19 October 2019.

[25] Moreno DJ Pino S Ríos Á Lopera F Ostos H Via M Genetic ancestry and susceptibility to late-onset Alzheimer Disease (LOAD) in the admixed Colombian population. Alzheimer Dis Assoc Disord. 2017;31:225-31. 10.1097/WAD.0000000000000195 28369008

[26] Warren LA Shi Q Young K Borenstein A Martiniuk A Prevalence and incidence of dementia among indigenous populations:a systematic review. Int Psychogeriatr. 2015;27:1959-70. . 10.1017/S1041610215000861 26088474

[27] de Souza-Talarico JN de Carvalho AP Brucki SM Nitrini R Ferretti-Rebustini RE Dementia and Cognitive Impairment Prevalence and Associated Factors in Indigenous Populations: A Systematic Review. Alzheimer Dis Assoc Disord. 2016;30:281-7. 10.1097/WAD.0000000000000140 26840546

[28] de Carvalho AP. The prevalence of cognitive impairment in elderly and adult indigenous populations. Thesis. São Paulo: Escola de Enfermagem Universidade de São Paulo, Brasil, 2016 (in Portuguese; English abstract). Available: https://www.teses.usp.br/teses/disponiveis/7/7139/tde-17052017-112020/publico/Dra_Anna_tese_11maio_format.pdf. Accessed: 19 October 2019.

[29] Grinberg LT Nitrini R Suemoto CK Ferretti-Rebustini REL Leite RE Farfel JM Prevalence of dementia subtypes in a developing country: a clinicopathological study. Clinics (São Paulo). 2013;68:1140-5. 10.6061/clinics/2013(08)13 24037011 PMC3752642

[30] Suemoto CK Ferretti-Rebustini RE Rodriguez RD Leite RE Soterio L Brucki SM Neuropathological diagnoses and clinical correlates in older adults in Brazil: A cross-sectional study. PLoS Med. 2017;14:e1002267. 10.1371/journal.pmed.1002267 28350821 PMC5369698

[31] Livingston G Sommerlad A Orgeta V Costafreda SG Huntley J Ames D Dementia prevention, intervention, and care. Lancet. 2017;390:2673-734. 10.1016/S0140-6736(17)31363-6 28735855

[32] Mukadam N Sommerlad A Huntley J Livingston G Population attributable fractions for risk factors for dementia in low-income and middle-income countries: an analysis using cross-sectional survey data. Lancet Glob Health. 2019;7:e596-603. 10.1016/S2214-109X(19)30074-9 31000129 PMC7617123

[33] Folstein MF Folstein SE McHugh PR “Mini-mental state”. A practical method for grading the cognitive state of patients for the clinician. J Psychiatr Res. 1975;12:189-98. 10.1016/0022-3956(75)90026-6 1202204

[34] Nasreddine ZS Phillips NA Bédirian V Charbonneau S Whitehead V Collin I The Montreal Cognitive Assessment, MoCA:a brief screening tool for mild cognitive impairment. J Am Geriatr Soc. 2005;53:695-9. 10.1111/j.1532-5415.2005.53221.x 15817019

[35] Lezak MD. Neuropsychological Assessment. Oxford: Oxford University Press; 1976.

[36] Mattis S. Mental state examination for organic mental syndromes in the elderly patient. In Bellak L, and Karasu TE (eds): Geriatric Psychiatry. New York: Grune & Straton; 1976.

[37] Mioshi E Dawson K Mitchell J Arnold R Hodges JR The Addenbrooke’s Cognitive Examination Revised (ACE-R):a brief cognitive test battery for dementia screening. Int J Geriatr Psychiatry. 2006;21:1078-85. 10.1002/gps.1610 16977673

[38] Morris JC Mohs RC Rogers H Fillenbaum G Heyman A Consortium to establish a registry for Alzheimer’s disease (CERAD) clinical and neuropsychological assessment of Alzheimer’s disease. Psychopharmacol Bull. 1988;24:641-52. 3249766

[39] de Wall C Wilson BA Baddeley AD The Extended Rivermead Behavioural Memory Test: a measure of everyday memory performance in normal adults. Memory. 1994;2:149-66. 10.1080/09658219408258942 7584289

[40] Wall JR Deshpande S MacNeil SE Lichtenberg PA The fuld object memory evaluation, a useful tool in the assessment of urban geriatric patients. Clin Gerontol. 1998;19:39-49. 10.1300/J018v19n01_04

[41] Della Sala S Kozlova I Stamate A Parra MA A transcultural cognitive marker of Alzheimer’s Disease. Int J Geriatr Psychiatry. 2018;33:849-56. 10.1002/gps.4610 27805729

[42] Storey JE Rowland JTJ Conforti DA Dickson HG The Rowland Universal Dementia Assessment Scale (RUDAS): a multicultural cognitive assessment scale. Int Psychogeriatr. 2004;16:13-31. 10.1017/S1041610204000043 15190994

[43] Ardila A Ostrosky-Solis F Rosselli M Gómez C Age-related cognitive decline during normal aging: the complex effect of education. Arch Clin Neuropsychol. 2000;15:495-513. 14590204

[44] Nitrini R Lefèvre BH Mathias SC Caramelli P Carrilho PE Sauaia N Neuropsychological tests of simple application for diagnosing]. [In Portugese]. Arq Neuropsiquiatr. 1994;52:457-65. 10.1590/S0004-282X1994000400001 7611936

[45] Nitrini R Caramelli P Porto CS Charchat-Fichman H Formigoni AP Carthery-Goulart MT Brief cognitive battery in the diagnosis of mild Alzheimer’s disease in subjects with medium and high levels of education. Dement Neuropsychol. 2007;1:32-6. 10.1590/S1980-57642008DN10100006 29213365 PMC5619381

[46] Baiyewu O Unverzagt FW Lane KA Gureje O Ogunniyi A Musick B The Stick Design test: a new measure of visuoconstructional ability. J Int Neuropsychol Soc. 2005;11:598-605. 10.1017/S135561770505071X 16212687

[47] Ortega LFV Aprahamian I Borges MK Cação JC Yassuda MS Screening for Alzheimer’s disease in low-educated or illiterate older adults in Brazil: a systematic review. Arq Neuropsiquiatr. 2019;77:279-88. 10.1590/0004-282x20190024 31090809

[48] Paddick SM Gray WK McGuire J Richardson J Dotchin C Walker RW Cognitive screening tools for identification of dementia in illiterate and low-educated older adults, a systematic review and meta-analysis. Int Psychogeriatr. 2017;29:897-929. 10.1017/S1041610216001976 28274299

[49] Pfeffer RI Kurosaki TT Harrah CH Jr Chance JM Filos S Measurement of functional activities in older adults in the community. J Gerontol. 1982;37:323-9. 10.1093/geronj/37.3.323 7069156

[50] Nitrini R Caramelli P Bottino CM Damasceno BP Brucki SM Anghinah R Diagnosis of Alzheimer’s disease in Brazil: cognitive and functional evaluation. Recommendations of the Scientific Department of Cognitive Neurology and Aging of the Brazilian Academy of Neurology. Arq Neuropsiquiatr. 2005;63:720-7. 10.1590/S0004-282X2005000400034 16172733

[51] Quiroga P Albala C Klaasen G Validation of a screening test for age associated cognitive impairment, in Chile. Rev Med Chil. 2004;132:467-78. 15382519 10.4067/s0034-98872004000400009

[52] Sánchez SS Abanto J Sanchez-Boluarte A Boluarte-Carbajal A Sanchez-Coronel D Custodio-Capuñay N Frequency and associated factors of amnestic mild cognitive impairment at four senior citizen clubs in Lima, Peru. Dement Neuropsychol. 2019;13:321-8. 10.1590/1980-57642018dn13-030009 31555405 PMC6753901

[53] Jorm AF Christensen H Henderson AS Jacomb PA Korten AE Mackinnon A Informant ratings of cognitive decline of elderly people: relationship to longitudinal change on cognitive tests. Age Ageing. 1996;25:126-9. 10.1093/ageing/25.2.125 8670540

[54] Galvin JE Roe CM Morris JC Evaluation of cognitive impairment in older adults: combining brief informant and performance measures. Arch Neurol. 2007;64:718-24. 10.1001/archneur.64.5.718 17502471

[55] Damin AE Nitrini R Brucki SMD Cognitive Change Questionnaire as a method for cognitive impairment screening. Dement Neuropsychol. 2015;9:237-44. 10.1590/1980-57642015dn93000005 29213967 PMC5619364

[56] Amado DK Brucki SMD Knowledge about Alzheimer’s disease in the Brazilian population. Arq Neuropsiquiatr. 2018;76:775-82. 10.1590/0004-282x20180106 30570022

[57] Lawlor B The local and global imperative to raise public awareness and knowledge about dementia. Arq Neuropsiquiatr. 2018;76:729-30. 10.1590/0004-282x20180118 30570014

[58] Gonzalez FJ Gaona C Quintero M Chavez CA Selga J Maestre GE Building capacity for dementia care in Latin America and the Caribbean. Dement Neuropsychol. 2014;8:310-6. 10.1590/S1980-57642014DN84000002 25932285 PMC4412169

[59] Winblad B Amouyel P Andrieu S Ballard C Brayne C Brodaty H Defeating Alzheimer’s disease and other dementias: a priority for European science and society. Lancet Neurol. 2016;15:455-532. 10.1016/S1474-4422(16)00062-4 26987701

[60] Jacinto AF Brucki S Porto CS Martins MA Nitrini R Detection of cognitive impairment in the elderly by general internists in Brazil. Clinics (São Paulo). 2011;66:1379-84. 10.1590/S1807-59322011000800012 21915487 PMC3161215

[61] Valcour VG Masaki H Curb JD Blanchette PL The detection of dementia in the primary care setting. Arch Intern Med. 2000;160:2964-8. 10.1001/archinte.160.19.2964 11041904

[62] Finkel SI Cognitive screening in the primary care setting: the role of physicians at the first point entry. Geriatrics. 2003;58:43-4. 12813873

[63] Ngandu T Lehtisalo J Solomon A Levälahti E Ahtiluoto S Antikainen R A two-year multidomain intervention of diet, exercise, cognitive training, and vascular risk monitoring versus control to prevent cognitive decline in at-risk elderly people (FINGER): a randomized controlled trial. Lancet. 2015;385:2255-63. 10.1016/S0140-6736(15)60461-5 25771249

[64] Kivipelto M Mangialasche F Ngandu T World Wide Fingers Network World Wide Fingers will advance dementia prevention. Lancet Neurol. 2018;17:27. 10.1016/S1474-4422(17)30431-3 29263003

[65] Jacinto AF Brucki SMD Porto CS Martins MA Nitrini R Screening of cognitive impairment by general internists using two simple instruments. Dement Neuropsychol. 2012;6:42-7. 10.1590/S1980-57642012DN06010007 29213771 PMC5619106

[66] Ranson JM Kuźma E Hamilton W Muniz-Terrera G Langa KM Llewellyn DJ Predictors of dementia misclassification when using brief cognitive assessments. Neurol Clin Pract. 2019;9:109-17. 10.1212/CPJ.0000000000000566 31041124 PMC6461420

[67] Allegri RF Butman J Arizaga RL Machnicki G Serrano C Taragano FE Economic impact of dementia in developing countries: an evaluation of costs of Alzheimer-type dementia in Argentina. Int Psychogeriatr. 2007;19:705-18. 10.1017/S1041610206003784 16870037

[68] Veras R Caldas C Dantas S Sancho L Sicsu B Motta L Demented elderly people living at home in Rio de Janeiro, Brazil: Evaluation of expenditure on care. Psychogeriatrics. 2008;8:88-95. 10.1111/j.1479-8301.2008.00237.x

[69] Ferretti C Sarti FM Nitrini R Ferreira FF Brucki SMD An assessment of direct and indirect costs of dementia in Brazil. PLoS One. 2018;13:e0193209. 10.1371/journal.pone.0193209 29494693 PMC5832239

[70] Hojman DA Duarte F Ruiz-Tagle J Budnich M Delgado C Slachevsky A The cost of dementia in an unequal country: The case of Chile. PLoS One. 2017;12:e0172204. 10.1371/journal.pone.0172204 28267795 PMC5340351

[71] Tapia-Muñoz T Slachevsky A León-Campos MO Madrid M Caqueo-Urízar A Rohde GC Predictors of unmet needs in Chilean older people with dementia: a cross-sectional study. BMC Geriatr. 2019;19:106. 10.1186/s12877-019-1131-1 30987587 PMC6466805

[72] Villalobos Dintrans P Informal caregivers in Chile: the equity dimension of an invisible burden. Health Policy Plan. 2019;34:792-9. 10.1093/heapol/czz120 31603492

[73] Custodio N Lira D Herrera-Perez E Del Prado LN Parodi J Guevara-Silva E Cost-of-illness study in a retrospective cohort of patients with dementia in Lima, Peru. Dement Neuropsychol. 2015;9:32-41. 10.1590/S1980-57642015DN91000006 29213939 PMC5618989

[74] Mayston R Lloyd-Sherlock P Gallardo S Wang H Huang Y Montes de Oca V A journey without maps-Understanding the costs of caring for dependent older people in Nigeria, China, Mexico and Peru. PLoS One. 2017;12:e0182360. 10.1371/journal.pone.0182360 28787029 PMC5546609

[75] Liu Z. Economic Costs of Dementia in Low and Middle Income Countries. Thesis. King’s College London, 2013. Available: https://kclpure.kcl.ac.uk/portal/en/theses/economic-costs-of-dementia-in-low-and-middle-income-countries(9d90e06c-022d-4db0-a877-e84f859531e4).html. Accessed: 19 October 2019.

[76] Wimo A Guerchet M Ali GC Wu YT Prina AM Winblad B The worldwide costs of dementia 2015 and comparisons with 2010. Alzheimers Dement. 2017;13:1-7. 10.1016/j.jalz.2016.07.150 27583652 PMC5232417

[77] Oliva-Moreno J Trapero-Bertran M Peña-Longobardo LM Del Pozo-Rubio R The valuation of informal care in cost-of-illness studies: A systematic review. Pharmacoeconomics. 2017;35:331-45. 10.1007/s40273-016-0468-y 27848219

[78] Acosta D Rottbeck R Rodriguez G Ferri CP Prince MJ The epidemiology of dependency among urban-dwelling older people in the Dominican Republic; a cross-sectional survey. BMC Public Health. 2008;8:285. 10.1186/1471-2458-8-285 18700967 PMC2551614

[79] Villalobos Dintrans P Informal caregivers in Chile: the equity dimension of an invisible burden. Health Policy Plan. 2019;34:792-9. 10.1093/heapol/czz120 31603492

[80] Harwood RH Sayer AA Hirschfeld M Current and future worldwide prevalence of dependency, its relationship to total population, and dependency ratios. Bull World Health Organ. 2004;82:251-8. 15259253 PMC2585969

[81] Garcia A Bucher-Maluschke JSN Pérez-Angarita DM Yushiara Emily VV Pereira FN Couple and family relationships in Latin American social comparative studies. Interpersona. 2016;109-124. 10.5964/ijpr.v10i2.259

